# Cardiac anomalies associated with Escobar syndrome: A case report and a review of the literature

**DOI:** 10.1097/MD.0000000000026687

**Published:** 2021-07-30

**Authors:** Naif Saad ALGhasab, Bandar Alshehri, Leen Abdullah Altamimi, Raghad Asaad Assiri, Loujain Ahmad AlYousef, Sulaman ALMesned, Fayez Saud ALreshidi, Suleiman M. Kharabsheh, Sara Abou Al-Saud, Waleed Alharbi

**Affiliations:** a Department of Internal Medicine, Medical College, Ha’il University, Ha’il, Saudi Arabia; b Adult Cardiology Department, Prince Sultan Cardiac Center, Riyadh, Saudi Arabia; c College of Medicine, King Saud University, Riyadh, Saudi Arabia; d College of Medicine, Al-Imam Mohammad Ibn Saud Islamic University, Riyadh, Saudi Arabia; e College of Medicine, King Saud bin Abdulaziz University for Health Sciences, Riyadh, Saudi Arabia; f Department of Surgery, Medical College, Qassim University, Buraydah, Saudi Arabia; g Department of Family and Community Medicine, College of Medicine, University of Hail, Hail, Saudi Arabia; h Department of Cardiovascular Disease, King Faisal Specialist Hospital and Research Centre, Riyadh, Saudi Arabia; i Department of Cardiac Science, College of Medicine, King Saud University, Riyadh, Saudi Arabia.

**Keywords:** cardiac anomalies, Escobar syndrome, isolated dextrocardia, situs ambiguous, situs inversus

## Abstract

**Rationale:**

Escobar syndrome (ES) is an autosomal recessive disorder. It is highly characterized by facial abnormalities, congenital diaphragmatic muscle weakness, myasthenic-like features, and skin pterygiums on multiple body legions. ES is a rare condition associated with many external and internal abnormalities. The internal malformations described in ES affect many organs including the heart, lungs, esophagus, liver, spleen, and intestine. The purpose of this paper is to explore the cardiac manifestations associated with ES.

**Patient concerns:**

A 3.5-year-old girl, who was born for double first cousins, was admitted to the hospital for neuromuscular evaluation of multiple congenital contractures.

**Diagnosis:**

The girl was diagnosed with ES and isolated dextrocardia which is a rare cardiac manifestation. However, to the best of our knowledge, no similar cases have been reported to date, and this case is thus believed to be very rare.

**Interventions:**

The patient underwent an operative intervention to correct the bilateral fixed flexion deformity at her knees which was related to the posterior bilateral fibrotic bands/pterygia.

**Outcomes:**

Post-operatively, complete knee extension was obtained, the patient was fitted with a cast and extension night splint. She was discharged alive and had no complications. The patient was followed regularly in the orthopedic clinic and had periodic physiotherapy sessions.

**Conclusions:**

ES and isolated dextrocardia concurrence in the presented case resulted from different pathogenic mechanisms. Our findings suggest that ES might be caused by dysfunction in the acetylcholine receptor throughout fetal life, which may have affected muscle strength and movement. Other cardiac conditions include hypoplastic left-sided heart, Hypertrophic cardiomyopathy, patent ductus arteriosus, and heterotaxia.

## 1 Introduction

Escobar syndrome (ES) is an autosomal recessive disorder that was first described by Bussiere nearly a century ago in South India.^[1]^ This condition is considered rare, and it is highly characterized by facial abnormalities, congenital diaphragmatic muscle weakness, myasthenic-like features, and skin pterygiums on multiple body legions (eg, cervical, antecubital, popliteal, interdigital, and on the neck) which have been frequently reported.^[2–4]^ Therefore, it was named as multiple pterygium syndrome by Gorlin later in 1976,^[5]^ then it got the final nomenclature as ES in 1982 after Escobar, a comprehensive report on this condition was published in 1978.^[6]^

ES is associated with many external and internal abnormalities such as congenital heart defects in some individuals, but this approach remains briefly addressed in the literature.^[7]^ The aim of this study is to present the clinical data of a patient with ES and isolated dextrocardia, with a review of relevant literature to explore the cardiac manifestations associated with ES.

## 2 Methods

The review of the literature was conducted utilizing PubMed, ScienceDirect, and Google Scholar databases to ensure all relevant studies on ES are included. Subject headings and medical subject heading terms, as well as different combinations of basic search terms on ES were used to search these databases, so all the conclusions are based on an all-inclusive knowledge base.

Data were extracted, and the quality was checked by three independent reviewers. All data were reviewed, summarized, compared, and clinical assessments were plotted. In addition, predetermined rules were set to provide a basis for including and excluding certain studies. Studies met inclusion criteria (i) all published scientific papers that focused on ES cases; (ii) systematic studies and medical case reports that were published in English and had a high level of evidence; and (iii) published between 1902 and 2019. Articles were excluded if a case was presented in another study.

### 2.1 Case presentation

A 3.5-year-old girl was referred to a tertiary care hospital in Riyadh, Saudi Arabia from one of the Ministry of Health Hospitals, for neuromuscular evaluation of congenital contractures on August 6, 2011. The patient's mother confirmed a full term of a normal pregnancy with normal fetal movements which is a sign that the fetus is growing in size and strength. Cesarean section was recommended for delivering the patient due to prolonged labor. Although there were no perinatal problems reported, the patient was born with contractures particularly affecting her fingers, knees, and feet. Over time, those contractures improved with periodic physiotherapy sessions particularly her thumbs and 4th and 5th fingers which were often flexed in the palm. According to her parents, the patient is cognitively intelligent, and both her speech and language are matching the 99th percentile. On the contrary to the negative progression of this condition, there was a significant improvement where she was able to crawl and stand on her knees at an early age. Moreover, she is not on any medications, has no known allergies, and has never been hospitalized nor had any surgery.

The patient is followed regularly by orthopedics for consideration of soft tissue release particularly with current talipes more obvious on the left side. She also has pterygium webbing of the knee joints limiting them from the extension. Whereas ES has an unknown incidence, it is more common among children from consanguineous relationships. Therefore, the family pedigree was checked, and it was found that the parents are double first-degree cousins, and they have 2 girls and 1 boy who are healthy with no similar family history reported.

Upon arrival to the hospital and after documenting the patient's medical history, physical examination showed the following: temperature 37.1°C, respiratory rate 20 breaths/min, pulse rate 96 beats/min, blood pressure 110/60 mmHg, and blood oxygen saturation 96%. Furthermore, weight was 9.5 kg, head circumference was 45 cm, and cranial nerve examination was unremarkable. She has, however, some distinct dysmorphic facial features (ie, triangular face with micrognathia and prominent forehead, narrow palpebral fissures, deep-set eyes, and low-set ear). There was no tongue fasciculation. On the neuromuscular assessment, she showed isometric strength around the shoulder, the elbows, and relatively preserved strength with finger flexion. The hands, however, showed mild deformity of the 4th and 5th fingers and the creases present with no obvious hypothenar muscle eminence. In the lower extremity, a clear thinning of the leg muscles was detected compared to the thigh associated with pterygium behind both knees limiting knees extension. In addition, the Achilles tendon shortening was noticed more on the left side with inversion, whereas limited ankle dorsiflexion appeared on the right side with weakness distally. Reflexes were difficult to elicit, but responses were +1 around the knees and the biceps level. Clinical examination of the patient's back showed subtle scoliosis but when pulled to a sitting position the head was lagging and the patient was not able to hold the head up for longer periods, however, she was not very cooperative according to her parents.

**Figure 1:**
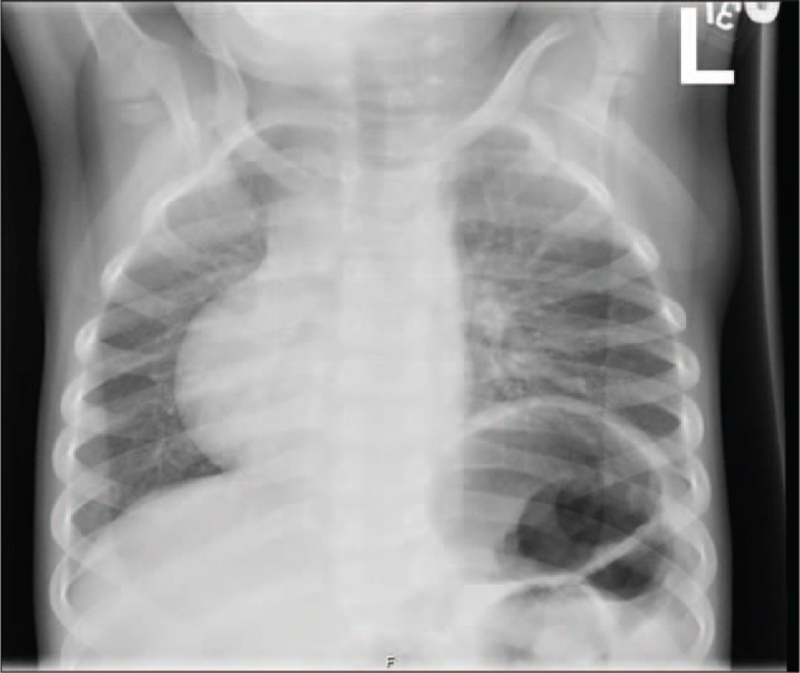
Anteroposterior radiograph of the chest from a 3-year-old girl shows dextrocardia with left-sided aortic arch and stomach. There is a gastric bubble with an elevated left hemidiaphragm.

**Figure 2:**
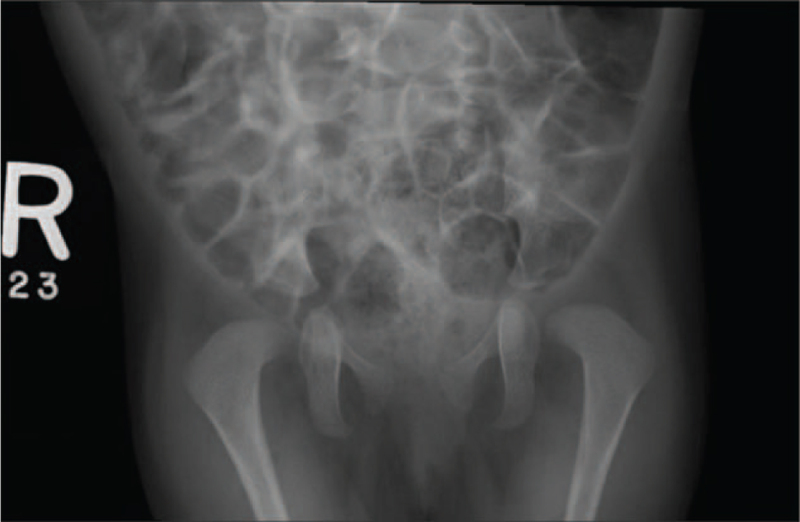
Anteroposterior radiograph of the pelvis from a 3-year-old girl shows the normal articulation of the proximal femurs with hip joints; however, there is slight breakage of the left Shenton line.

**Figure 3:**
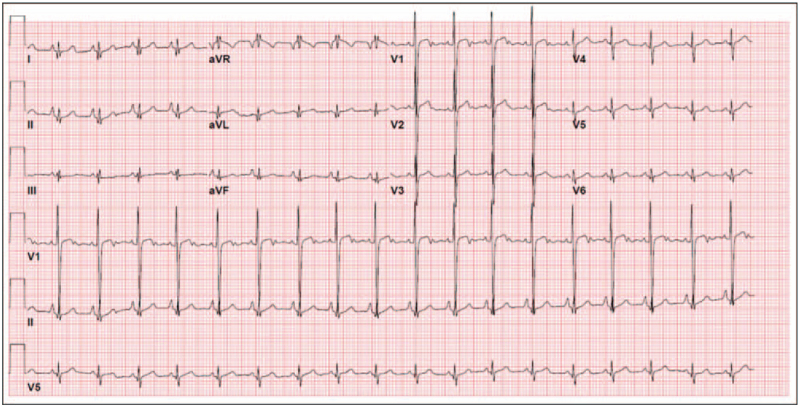
A 12-lead electrocardiogram from a 3-year-old girl with dextrocardia. Results demonstrate a normal sinus rhythm, a positive QRS complex in lead aVR, and a positive R-wave in leads V_1_ to V_6_. However, the normal progression of the R wave is not observed, the amplitude of R wave in lead V_1_ is more than the amplitude of R wave in lead V_6_.

Complete blood count and routine chemistry profiles were in normal ranges. White blood cells and differential count (lymphocytes) study was also normal, including Human leukocyte antigen, blastogenesis normal responses, ratios, CD4/CD8 ratios, Antihuman B lymphocyte serum, and T-helper cells. At the genomic level, an array comparative genomic hybridization using microarray was used to detect any chromosomal copy number changes on the genome which appeared normal without imbalance. Also, the patient's Karyotype was normal. The skeletal survey showed normal bone density, dextrocardia with left-sided aortic arch and stomach, and no signs of skeletal dysplasia (Fig. 1). The anteroposterior radiographs of the pelvis showed normal articulation with slight breakage of the Shenton line (Fig. 2). Abdominal ultrasound showed normal liver, gallbladder, spleen, and pancreas. Multiple bilateral renal cysts were observed in both right and left kidneys measuring 4.9 and 5.7 cm, respectively. Knees magnetic resonance imaging (without contrast) showed bilateral knee flexion contracture deformities of the posterior fibrotic bands/pterygia with the more posterior location of the neuron bundle. Echocardiography showed dextrocardia with situs solitus and persistent left superior vena cava to the coronary sinus. The very small patent ductus arteriosus producing right-to-left shunt and patent foramen ovale causing left-to-right shunt were confirmed. Electrocardiogram showed normal sinus rhythm with dextrocardia (Fig. 3).

The patient was followed in the orthopedic clinic and underwent an operative intervention to correct the bilateral fixed flexion deformity at her knees which was related to the posterior bilateral fibrotic bands/pterygia. Surgical realignment was rendered, and a complete extension was obtained. Post-operative, the patient used cast and extension night splint. During follow-up, the patient had no complications, and she was compliant to the regular orthopedics appointments and the periodic physiotherapy sessions.

### 2.2 Literature review

The authors conducted an extensive literature search, out of 160 papers only 9 articles were included with 9 patients that met the inclusion criteria. Patients’ clinical manifestations are presented in Table 1. Clinical characteristics of our patient with a Barrera et al article presented in Table 2. Cardiac anomalies associated with ES are presented in Table 3.

**Table 1:** Detailed review of Escobar syndrome cases in the literature with cardiac anomalies.

	Name of study	Number of cases	Cardiac manifestations	Non-cardiac manifestations	References
1	Continuous fetal head flexion as a marker for prenatal diagnosis of lethal multiple pterygium syndrome: a case report	1	Absent end-diastolic flow of the umbilical artery, reversed flow of the ductus venosus, and mild tricuspid. Regurgitation and pericardial effusion.	Upper limbs: neck contracture and micrognathia, head flexion, elbow could not maximally extend.Lower limbs: knee joints could not maximally extend,Craniofacial: low-set earsOthers: pterygium of all joints, fetal ascites, pleural effusion and pulmonary hypoplasia, and intestinal malrotation.	Hasegawa et al ^[18]^ (2017)
2	Myopathies associated with β-tropomyosin mutations	1	Vesicular ventricular block with right bundle block and left anterior hemiblock	Others: severe hypotonia and arthrogryposis	Tajsharghi et al ^[19]^ (2012)
3	Anesthetic management of a pregnant patient with multiple pterygium syndrome (Escobar type).	1	ASD	Upper limbs: webbing of the neckLower limbs: nonCraniofacial: micrognathia, repaired cleft palate, low nasal bridge, small naresOthers: pectus carinatus with thoracic kyphoscoliosis.	Stoll et al ^[20]^ (2012)
4	Familial occurrence of multiple pterygium syndrome: expression in a heterozygote of the recessive form or variability of the dominant form?	1	ASD with a left-right shunt that closed spontaneously.	Upper limbs: flexion contractures of elbows and fingers, neck, axillae, marked camptodactyly and mild syndactyly of the 2nd, 3^rd^, and 4th fingers and clinodactyly of the 5th finger.Lower limbs: right popliteal fossa contractureCraniofacial: Pterygium colli, short palpebral fissures, inner canthal folds, hypertelorism, ptosis of eyelids, wide nasal root, small mouth with down-turning corners, micrognathia, low-set and posteriorly rotated ears and long eyelashes.Others: pectus excavatum with widely spaced nipples	Prontera et al ^[21]^ (2006)
5	Sonographic features of lethal multiple pterygium syndrome at 14 weeks.	1	Suspected Hypoplastic left heart.	Upper limbs: Pterygia: chin to sternum, axillaeLower limbs: Pterygia: antecubital, popliteal regions and bilateral rocker-bottom feet, clubfeetCraniofacial: micrognathia, down slanting palpebral fissures and low-set malformed earsOthers: non	Chen et al ^[22]^ (2005)
6	Multicore myopathy associated with multiple pterygium syndrome and hypertrophic cardiomyopathy	1	Hypertrophic cardiomyopathy (HCM)	Others: multicore myopathy	Ohkubo et al ^[23]^ (1996)
7	Syndrome of multiple pterygia, camptodactyly, facial anomalies, hypoplastic lungs and heart, cystic hygroma, and skeletal anomalies: delineation of a new entity and review of lethal forms of multiple pterygium syndrome.	1	Cardiac hypoplasia	Upper limbs: short neckLower limbs:Craniofacial: abnormal facial appearance with hypertelorism, markedly flattened nasal bridge with hypoplastic nasal alae, cleft palate, micrognathia, apparently low-set malformed earsOthers: cystic hygroma at the back of the neck and head, pulmonary hypoplasia, flexion contractures of multiple joints, small chest, hydrops and multiple pterygia involving chin, sternum, cervical, axillary, antecubital, and crural	Chen et al ^[24]^ (1984)
8	Lethal multiple pterygium syndrome: report of a case with neurological anomalies.	1	Cardiac hypoplasia	Craniofacial: cystic hygroma, hydrops, facial anomalies, microcephaly, brain immaturityOthers: joint contractures, muscle hypoplasia, severe cerebellar and pontine hypoplasia with the absence of the pyramidal tracts, pulmonary hypoplasia, growth retardation	Spearritt et al ^[25]^ (1993)
9	Limb pterygium syndromes: a review and report of eleven patients.	1	Cardiac hypoplasia	Upper limbs: finger contractures, syndactyly, webbing and contractures of the elbows, neck, and fingersLower limbs: rocker-bottom feet, webbing and contractures of the knees, calcaneovalgus deformity of the feetCraniofacial: ptosis, antimongoloid slant of palpebral fissures, epicanthal folds, highly arched palate, hypertelorism, flat nose, antimongoloid slant of palpebral fissures, apparently low-set ears, cleft palate.Others: multiple joint webs, scoliosis, and short stature, bilateral pulmonary hypoplasia, the absence of the appendix, and attenuation of the ascending and transverse colon.	Hall et al ^[14]^ (1982)

**Table 2:** Clinical characteristic of AlGhasab et al case and Barrera et al case.

ES phenotype characteristics	Case 1 (Barrera et al)^[8]^ (2014)	Case 2 AlGhasab et al (2011)
Perinatal		
Conductive deafness	−	−
Respiratory distress	+	−
Reduced facial movements	+	−
Reduced fetal movement	−	−
Growth retardation	+	−
Short stature	−	−
Facial dimorphism		
Long face	−	−
Ptosis	+	−
Hypertelorism	+	−
Downslanting palpebral fissures	−	−
Epicantal folds	−	−
Enlarged corneal nerves	−	−
Long philtrum	+	+
Small mouth	+	−
Down–turned corners of mouth	+	−
High-arched palate	−	−
Cleft palate	−	−
Lingual cochlearis	−	−
Furrowed tongue	−	−
Micrognathia	−	+
Retrognathism	+	−
Low set ears	+	−
Pterygia of the:		
Neck	+	−
Axillae	+	−
Antecubital	+	−
Popliteal	+	+
Fingers	−	+
Intercrural	+	−
Hypoplasic nipples	+	−
Cardiovascular anomalies		
Dilated root of the aorta	−	−
Aortic regurgitation	−	−
Small heart	+	−
Bilateral pulmonary hypoplasia	+	−
Gastrointestinal anomalies		
Absence of the appendix	−	−
Attenuation of the ascending and transverse colon.	−	−
Abdominopelvic area defects		
Wide umbilical area	−	−
Hypoplasia of the periumbilical skin	−	−
Inguinal hernia	−	−
Genitalia anomalies		
Cryptorchidism	+	−
Small penis	+	−
Small scrotum	+	−
Aplasia, hypoplasia or absent of the labia majora	NA	−
Small clitoris	NA	−
Osteoarticular anomalies		
Arthrogryposis multiplex congenital	+	+
Fusion of cervical vertebrae	+	−
Scoliosis or lordosis	−	+
Rib or vertebral anomalies	+	−
Congenital hip dislocation	−	−
Camptodactyly	+	−
Soft tissue syndactyly of fingers	+	−
Talipes equinovarus	+	+
Rocker-bottom feet	−	−
Vertical talus	−	−
Muscular anomalies		
Defects in the abdominal muscles	−	−
Sever muscular atrophy	−	−

**Table 3:** Cardiac anomalies associated with Escobar syndrome (literature review).

Cardiac anomalies
Cardiac hypoplasia
Hypertrophic cardiomyopathy
Hypoplastic left-sided heart
ASD with a left to right shunt
PDA
PFO
Dextrocardia with situs solitus
Heterotaxia (left isomerism)

## 3 Discussion

ES is a rare condition that presents with a wide clinical expression. The main clinical characteristics are detailed in (Table 2) with comparison to patients from Barrera et al^[8]^ For ES genetic disorder, several aspects of identity have been explored, but many questions remain. So far, it has not been clear exactly how gene mutation in the cholinergic receptor nicotinic gamma subunit (CHRNG) could lead to this disease.^[9]^

The mammalian muscle-type acetylcholine receptor (AChR) is a transmembrane pentameric glycoprotein with 2 alpha subunits, 1 beta, 1 delta, and 1 epsilon (in adult skeletal muscle) or gamma (in fetal and denervated muscle) subunit.^[9]^ The gamma subunit of AChR plays a significant role in neuromuscular organogenesis and ligand binding and disruption of gamma subunit expression prevent the correct localization of the receptor in cell membranes which are highly suggested to affect muscle contractures seen in the disease.^[9]^ Mild alteration in CHRNG replacing the gamma subunit to epsilon is believed to be responsible for the contractures seen in ES, but yet the exact mechanism is still unknown. However, the complete or severe disruption of fetal AChR causes lethal multiple pterygium syndrome.^[10]^

The known internal malformations described in ES affect many organs including the heart, lungs, esophagus, liver, spleen, and intestine. Our patient phenotype represents a mild non-lethal form of multiple pterygium syndrome due to facial dysmorphism, multiple pterygia, arthrogryposis, and bone anomalies, resulting in a clear diagnosis of ES. Our patient presented with a classical phenotype of ES but with some unique characteristics that include internal abnormalities such as isolated dextrocardia (situs solitus).

A review of other varieties of cardiac anomalies associated with ES is shown in detail in Table 3. Dextrocardia was reported as heterotaxia with left isomerism in a single case report with ES,^[8]^ in contrast to the current case, which presented with isolated dextrocardia. It has been proposed over the past years that the lack of migration can become the source of the embryological origin of dextrocardia, which may share a common origin with ES.^[9]^ At day 22 of gestation, the primitive cardiac loop usually bends to the right side and during cardiac septation (which lasts over the following 10–12 days) the apex of the heart gradually moves from the right side of the thorax to the left side of the chest pointing leftward.^[11]^ Similar mechanisms were suggested in ES as the absence of active fetal movements (akinesia). Fetal akinesia can start on the 8th week and lasts over for 3 weeks which may lead to the absence or defective musculoskeletal development including joints.^[12]^ As a result, an assumption can be made that dextrocardia and ES has a common migration problem that may affect the child during the 2nd and 3rd week of pregnancy.

In addition to the webbing of skin (pterygium) and the lack of muscle movement in ES, internal organs are affected.^[2,8,12,13]^ Several cases were associated with limb pterygium, contractures, bilateral pulmonary hypoplasia, small heart, the absence of the appendix, esophageal fistula, and abnormal ascending and/or transverse colon.^[13,14]^ Other uncommon features may involve the bone as hypoplasia of the humerus, the absence of the radius, ulna, hand, both fibula bones, and hypoplasia of sacrum.^[14]^ The general state of hypoplasia in the kidney, asymmetry of the body, and some other parts were addressed.^[14]^ Lethal conditions usually involve cardiac and lung hypoplasia which may end up with death.^[15]^

It has been suggested that fetal edema could play a crucial role in producing some of ES abnormalities such as pterygia, lung and gut hypoplasia, bone fusion, ocular hypertelorism, and cleft palate which might be a causative factor in dextrocardia.^[8]^ The attainable mechanism proposed postulates extracellular pressure increment originated by the edema impacts the normal development of embryonic tissues.^[8]^ Furthermore, it has been recently demonstrated that CHRNG homozygous mutation is responsible for the arthrogryposis multiplex congenital observed in ES.^[9]^ CHRNG mutations result in AChR dysfunction in fetal life reducing prenatal muscle strength and movement. Complete or severe functional disruption of fetal AChR is lethal and leads to multiple pterygium syndrome, whereas milder alteration could lead to fetal hypokinesia with congenital contractures or a myasthenic syndrome later in life.^[13,15,16]^

Familial occurrences have been reported in some cases of ES suggesting autosomal dominant, recessive, and X-linked patterns of inheritance.^[13,17]^ However, autosomal recessive inheritance appears to be the most common way of inheritance, and consanguinity has been associated with ES, a situation that is addressed in the case reported here.

Table 1 shows a detailed review of ES cases since its discovery to 2019, a total of 9 cases were investigated and cardiac and non-cardiac anomalies were documented.

## 4 Conclusion

In conclusion, ES and isolated dextrocardia concurrence resulted from different pathogenic mechanisms. ES might be caused by dysfunction in the AChR throughout fetal life, which may have affected muscle strength and movement. Dysfunction of left-right axis determination during early embryogenic development can cause dextrocardia. This report highlights the fact that ES may present with a clinical combination of dextrocardia and other spectra of anomalies that may be associated with this condition.

Finally, the authors hope that this article enlightens the gap of those special groups of patients as we try to change clinical practice with a better and new way of identification for such a condition.

## Acknowledgments

The authors didn’t have any writing assistance.

## Author contributions

The final draft is approved by all the authors.

**Conceptualization:** Naif Saad ALGhasab

**Data curation:** Leen Abdullah Altamimi, Raghad Asaad Assiri, Loujain Ahmad AlYousef

**Formal analysis:** Suleiman M. Kharabsheh, Sara Abou Al-Saud, Waleed Alharbi.

**Methodology and data analysis:** Suleiman M. Kharabsheh, Sara Abou Al-Saud, Waleed Alharbi

**Methodology:** Suleiman M. Kharabsheh, Sara Abou Al-Saud, Waleed Alharbi.

**Project administration:** Leen Abdullah Altamimi.

**Supervision:** Naif Saad ALGhasab

**Writing – original draft:** Naif Saad ALGhasab, Leen Abdullah Altamimi, Raghad Asaad Assiri, Loujain Ahmad AlYousef

**Writing – review & editing:** Naif Saad ALGhasab, Bandar Alshehri, Leen Abdullah Altamimi, Sulaman ALMesned, Fayez Saud ALreshidi.

## Correction

When originally published, Sulaman ALMesned's name appeared incorrectly as Sulaiman ALMesned and has since been corrected.
